# An ectopic thymoma arising in the middle mediastinum that was difficult to distinguish from a lymph node metastasis

**DOI:** 10.1186/s40792-021-01258-9

**Published:** 2021-08-03

**Authors:** Michiko Fukahori, Naoko Kimura, Yoshihiro Miyauchi, Kazuhiko Hirano, Kohei Morimoto, Miyuki Takahashi, Ayaka Ueda, Sayano Okazaki, Keisuke Taguchi, Yu Tsukahara, Sakurako Hattori, Yuki Suematsu, Masahiro Yan, Nobuhisa Teranishi, Kazuhiko Wakabayashi, Yutaka Itoh

**Affiliations:** 1 Department of Gastroenterological and Mammary Surgery, National Hospital Organization Disaster Medical Center, 3256 Midori-cho, Tachikawa-shi, Tokyo, 190-0014 Japan; 2 Department of Respiratory Surgery, National Hospital Organization Disaster Medical Center, 3256 Midori-cho, Tachikawa-shi, Tokyo, 190-0014 Japan; 3 Department of Pathology, National Hospital Organization Disaster Medical Center, 3256 Midori-cho, Tachikawa-shi, Tokyo, 190-0014 Japan; 4 Department of Diagnostic Radiology, National Hospital Organization Disaster Medical Center, 3256 Midori-cho, Tachikawa-shi, Tokyo, 190-0014 Japan

**Keywords:** Ectopic thymoma, Mediastinal lymph node metastasis, Dorsal innominate vein and superior vena cava in the parabronchial region

## Abstract

**Background:**

Ectopic thymomas often occur in the upper mediastinum; however, they rarely arise in the middle mediastinum, especially on the dorsal side of the innominate vein and superior vena cava in the peribronchial region.

**Case presentation:**

Six years prior, a 27-year-old female presented to our department and was diagnosed with locally advanced left breast cancer. First, we administered chemotherapy including an anti-human epidermal growth factor receptor 2 antibody. The size of the tumor was markedly reduced, and a radical operation involving mastectomy and axillary lymph node dissection was then performed. The patient underwent radiotherapy after the mastectomy, followed by trastuzumab therapy; she continued to receive endocrine therapy thereafter. She underwent computed tomography once a year after the surgery, and a nodule in the middle mediastinum on the dorsal side of the innominate vein and superior vena cava in the parabronchial region was detected at 4 years. We speculated that the nodule was a solitary mediastinal lymph node metastasis from her breast cancer; therefore, we performed thoracoscopic resection of the tumor. We diagnosed the tumor as a thymoma. Currently, the patient visits our hospital to receive continuous hormone therapy for her breast cancer, and the latest computed tomography scan demonstrated no metastases from or recurrence of her breast cancer or thymoma.

**Conclusions:**

We report a case of ectopic thymoma in the middle mediastinum. The tumor, which was detected during systemic therapy for locally advanced breast cancer, was located on the dorsal side of the innominate vein and superior vena cava in the parabronchial region and was indistinguishable from a lymph node metastasis from breast cancer.

## Background

Ectopic thymomas often occur in the upper mediastinum; however, they rarely arise in the middle mediastinum, especially on the dorsal side of the innominate vein and superior vena cava in the parabronchial region. Here, we report a case of ectopic thymoma that was indistinguishable from mediastinal lymph node metastasis from breast cancer.

## Case presentation

Six years prior, a 27-year-old female presented to our department with a large mass (diameter: approximately 70 mm) in her left breast and was diagnosed with locally advanced cancer of the left breast (T4bN1M0, stage IIIB, Union for International Cancer Control [UICC] classification, 7th edition). First, we administered an anthracycline-based therapy, followed by a taxane-based drug and an anti-human epidermal growth factor receptor 2 (HER2) antibody. The size of the tumor was markedly reduced, and a radical operation involving mastectomy and axillary lymph node dissection was then performed. Microscopic examination revealed scirrhous cancer in the left breast. The tumor [T4bN0(0/9), stage IIIB, estrogen receptor status: positive, progesterone receptor status: positive, HER2 receptor status: strongly positive, and Ki-67 index: 30%] measured approximately 10 mm in diameter. The patient underwent radiotherapy after the mastectomy, followed by trastuzumab (HER2-targeted) therapy; she continued to receive endocrine therapy thereafter.

**Fig. 1:**
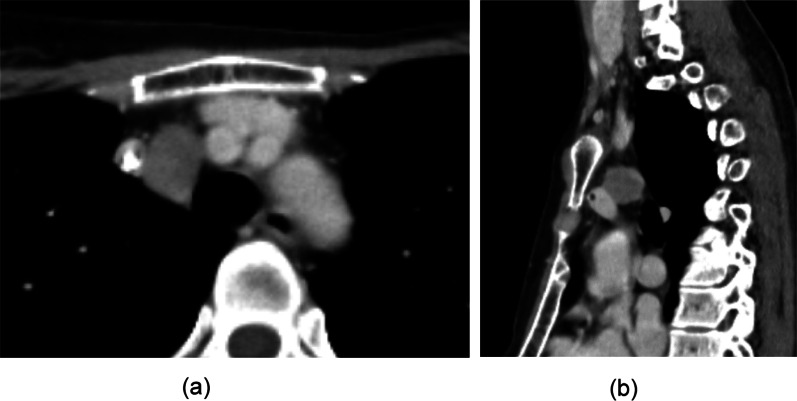
A chest CT scan obtained at 4 years after the patient's breast surgery. A mediastinal nodule (diameter: 23 mm) was detected on the horizontal (**a**) and sagittal (**b**) views

**Fig. 2:**
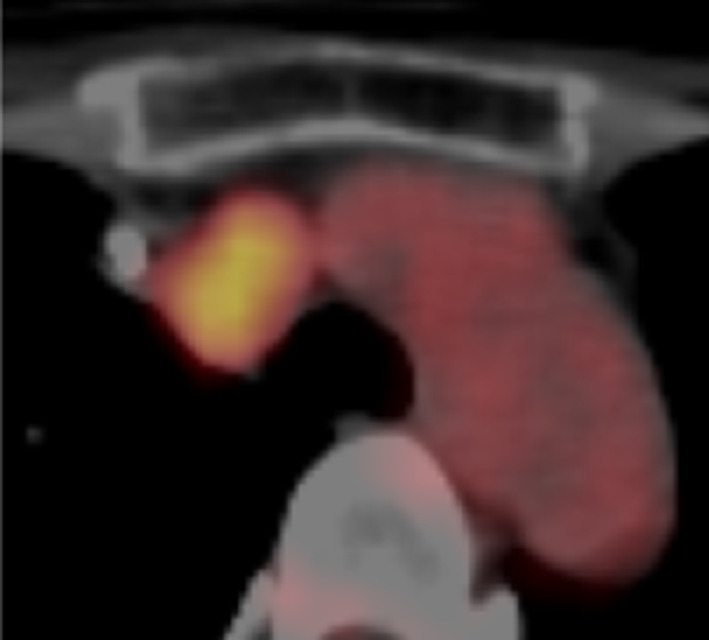
^18^F-FDG-PET/CT. The nodule was the only positive area in the whole body (SUVmax: 4.71)

**Fig. 3:**
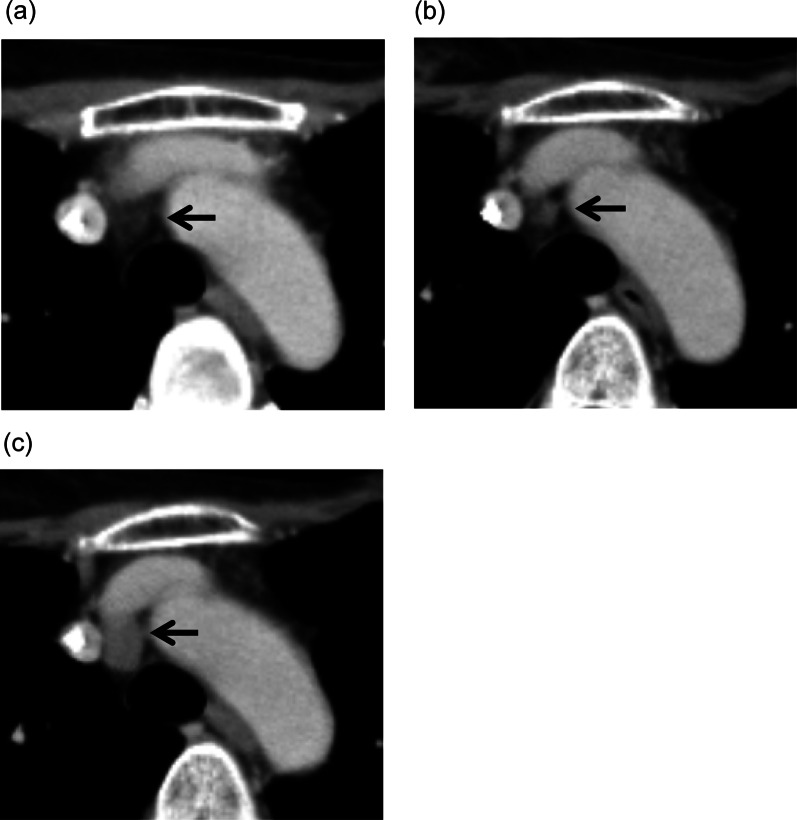
Chest CT scans obtained in the **a** first year, **b** second year, and **c** third year after surgery. The scans showed that the mediastinal nodule had been gradually growing for 3 years

**Fig. 4:**
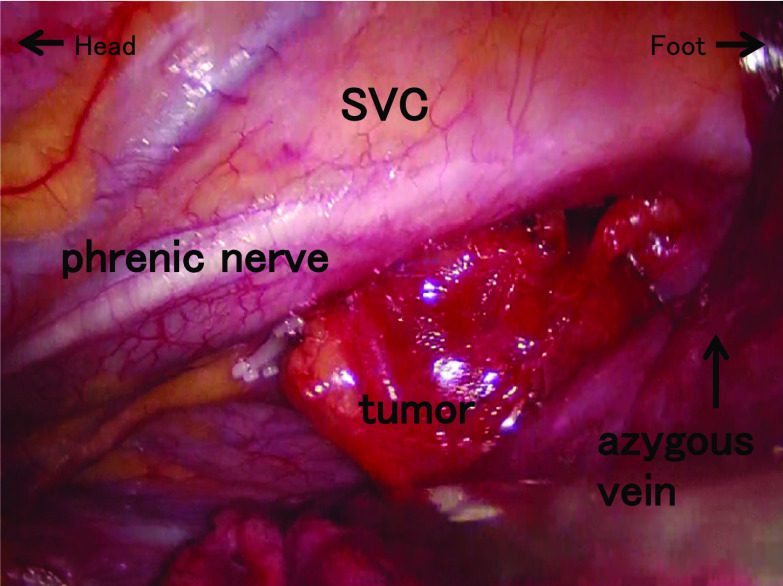
An intraoperative image. The image shows a tumor in the middle mediastinum

She underwent computed tomography (CT) once a year after undergoing surgery, and a mediastinal nodule measuring 23 mm in diameter was detected at 4 years (Fig. 1), which was the only positive site detected on ^18^F-FDG-PET/CT (maximum standardized uptake value [SUVmax]: 4.71) (Fig. 2). A retrospective review of previous CT scans revealed that the nodule had been gradually growing for 3 years (Fig. 3). The patient had no symptoms or abnormal laboratory data, including abnormal tumor marker levels. The anti-acetylcholine receptor antibody level was within normal limits. As we speculated that the nodule was a solitary mediastinal lymph node metastasis from her breast cancer, we performed thoracoscopic resection of the tumor approaching the right thoracic cavity (Fig. 4); the tumor had a capsule but had not invaded and was not attached to adjacent structures, and complete extirpation of the tumor was achieved.

**Fig. 5:**
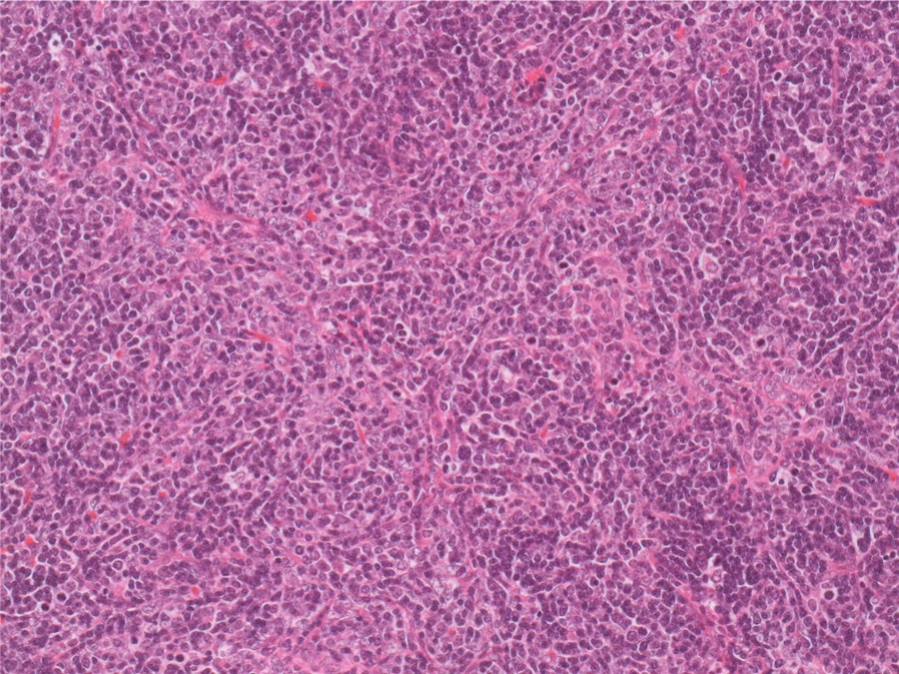
Pathological examination. Pathological examination showed that the tumor was a thymoma. The nodule was a lobular tumor containing many T lymphocytes and some thymic epithelial cells and was covered by a fibrous capsule, which had been partially invaded by tumor cells. Thymic gland tissue was found in the surrounding fat tissue. Hematoxylin and eosin staining, × 40

On microscopic examination, the nodule displayed a lobulated shape and contained many T lymphocytes and some thymic epithelial cells covered by a fibrous capsule. Local invasion of the tumor cells into the capsule was seen, and thymic gland tissue was found in the surrounding fat tissue. We diagnosed the tumor as a thymoma (World Health Organization [WHO] classification 2015 type B1 [1]; Masaoka stage II [2]; and T1aN0M0, stage I, according to the UICC-TNM 8th classification [3]) (Fig. 5).

We did not perform a supplemental total thymectomy, but decided to carry out CT frequently. Currently, the patient comes to our hospital to receive continuous hormone therapy for her breast cancer, and the latest CT scan (at one year after the tumorectomy) showed no metastases from or recurrence of her breast cancer or thymoma.

## Discussion

Thymoma is the most common anterior mediastinal tumor, accounting for up to 50% of all anterior mediastinal masses [4]. The WHO histological classification system for thymic epithelial tumors was revised in 2015 [1] and divides thymomas into 5 subtypes, thymoma types A, AB, B1, B2, and B3, based on the morphology of epithelial cells and the lymphocyte-to-epithelial cell ratio [5]. The Masaoka staging system is the most popular staging system [2], and preoperative identification of the histological subtype and tumor stage has important implications for the management, strategy, and prognosis of thymic epithelial tumors [5].

Thymomas often occur in the anterosuperior mediastinum [6]. On the other hand, ectopic thymomas are considered to arise from thymic tissue that has failed to migrate into the anterosuperior mediastinum [7], accounting for only 4% of all thymomas [7], 8]. Weissferdt et al. [6] reported that the incidence of ectopic thymomas was highest in the cervical region (45%), followed by the lungs (20%) and pleura (13%); other reported sites include the thyroid gland (6%), pericardium (7%), and middle/posterior mediastinum (4%). Wu et al. examined 114 cases of ectopic thymoma and found that the most common sites were the neck (43 cases), thyroid gland (17 cases), and lungs (11 cases), followed by the posterior mediastinum (8 cases) and middle mediastinum (6 cases) [8]. In addition, Hino et al. [9] reported a rare case of invasive ectopic thymoma in the thyroid and anterior mediastinum, and Yajima et al. [7] evaluated 13 cases of ectopic paratracheal thymoma of the middle mediastinum, finding them to be extremely rare. Our case is also rare, as the tumor was located in the middle mediastinum on the dorsal side of the innominate vein, adjacent to the superior vena cava, in the peribronchial region.

During embryonic development, thymic tissue originates from the third or fourth pair of pharyngeal sacs, and the bilateral lobes are located in the anterior superior mediastinum by the 8th week [3]. An ectopic thymus can occur due to the failure of migration during embryological development [8]. Li et al. [10] summarized ten studies and reported common locations of ectopic thymic tissue in mediastinal fat in patients with myasthenia gravis. Moreover, the presence of ectopic thymic tissue was investigated in different anatomical locations in 882 patients, with 509 (58%) having at least one positive location. The most common sites were anterior mediastinal fat in 293 (33.2%), pericardiophrenic angles in 120 (13.6%), the aortopulmonary window in 92 (10.4%), the cervical region (pretracheal fat) in 66 (7.5%), lateral to phrenic nerves in 34 (3.9%), the aortocaval groove in 14 (1.6%), and behind the innominate vein in 6 (0.7%). Although that report was based on retrospective evidence from patients with myasthenia gravis, it is very important to recognize the more extended localization of ectopic thymic tissue where ectopic thymoma can arise; indeed, such information might lead to diagnosis of a nodule as a thymoma.

Regarding breast cancer, Tsuji et al. retrospectively analyzed data for 252 recurrent breast cancer patients who were treated at their institution and reported that > 80% experienced recurrence in a single organ at the first recurrence; 40% experienced soft-tissue recurrence [11], even though breast cancer seldom causes solitary mediastinal lymph node metastases. In the current case, the fact that the nodule had been growing gradually for a few years without any other metastasis is an atypical course of a breast cancer lymph node metastasis.

Regarding procedures to diagnose a mass in a paratracheal lesion, endobronchial ultrasound-guided transbronchial needle aspiration (EBUS-TBNA) is less invasive than operations such as VATS. Argento et al. [12] assessed 64 cases and concluded that EBUS-TBNA is a useful tool for evaluating mediastinal lymphadenopathy in patients with a history of breast cancer and can provide information on the concordance of receptor status between the primary tumor and metastatic sites in the thorax. These authors reported breast malignancy in 33 (52%) patients, 23 (70%) of whom had sufficient samples to evaluate hormone receptors, such as estrogen, progesterone and human epidermal growth factor receptor 2 status. Although specimens obtained from EBUS-TBNA are so small that it has been considered difficult to diagnose thymoma on this basis, Yoshida et al. [13] reported two cases of thymomas diagnosed by EBUS-TBNA. Furthermore, Yajima et al. [7] stated that EBUS-TBNA may be useful for diagnosing a paratracheal tumor. EBUS-TBNA might have been considered an option in the present case, but we decided to resect the tumor as a diagnostic treatment for complete resection of the nodule because of technical limitations in our institution.

In the National Comprehensive Cancer Network (NCCN) medical care guidelines [14], total thymectomy and complete resection of the tumor are recommended for stage 1 and 2 thymomas. In the current case, we did not perform total thymectomy and decided to follow-up with the patient frequently because we were able to resect the tumor completely with adequate surgical margins, and the tumor was located away from the other tissue in the anterior mediastinum. Indeed, although no surgical procedures for the treatment of ectopic thymoma have been established, Yajima et al. concluded that simple resection of ectopic thymomas might be sufficient for patients without myasthenia gravis [7].

## Conclusions

We report a case of ectopic thymoma in the middle mediastinum. The tumor, which was detected during systemic therapy for locally advanced breast cancer, was located on the dorsal side of the innominate vein and superior vena cava in the peribronchial region and was indistinguishable from a lymph node metastasis from breast cancer. We should take into account the possibility that a solitary mediastinal nodule can be an ectopic thymoma, even during systematic therapy for breast cancer.

## Data Availability

The datasets supporting the conclusions of this article are included within the article.

## Abbreviations

^18^F-FDG-PET: ^18^Fluorodeoxyglucose positron emission tomography
